# Yiqi Daozhi Formula Reduces the M1 Polarization of Macrophages in Mice With Postoperative Ileus Through Mediating Glycolysis Metabolism

**DOI:** 10.1002/iid3.70353

**Published:** 2026-02-12

**Authors:** Wang Gang, Zhao Xuan, Wang Ye, Yi Chen, Yu Jing, Zhang Tianle, Shao Mingyue, Tao Yuewei, Jiang Zhiwei

**Affiliations:** ^1^ Affiliated Hospital of Nanjing University of Chinese Medicine Nanjing Jiangsu China; ^2^ First Clinical Medical College Nanjing University of Chinese Medicine Nanjing Jiangsu China; ^3^ School of Medicine University of Dundee, Ninewells Hospital Dundee UK

**Keywords:** M1 polarization, macrophage, postoperative ileus, Yiqi Daozhi Decoction

## Abstract

**Background:**

Postoperative ileus (POI) represents a disorder of gastrointestinal function following surgical procedures, characterized by a multifaceted etiology. There is an urgent clinical need to search for treatment regimens that improve the therapeutic effect for POI. Current research indicates that Traditional Chinese Medicine (TCM) demonstrates significant efficacy in treating POI. Our research was conducted to delve into the precise therapeutic action of Yiqi Daozhi Formula (YQDZF) in the treatment of POI.

**Methods:**

In our study, H&E staining and carmine detection were employed to assess the gastrointestinal functionality in the POI mouse model. ELISA was used to detect levels of MPO, lactic acid, inflammatory factors, and glycolysis. Flow cytometry and qRT‐PCR were employed to examine the levels and expression of macrophage phenotypic markers. Western blotting was used to detect the expression of glycolysis‐ and AKT/NF‐kB/HIF‐1α signaling pathway‐related proteins. Cycloheximide method and MG‐132 method were used to detect the stability of AKT protein.

**Results:**

After making the comparative analysis with the Control group, the POI group mice exhibited pronounced gastrointestinal dysfunction, which was mitigated by treatment with YQDZF. *In vivo* experiments confirmed that YQDZF treatment markedly decreased M1 macrophage polarization and inhibited the glycolytic pathway. Cellular experiments demonstrated that this therapeutic approach was related to the AKT/NF‐kB/HIF‐1α signaling pathway in macrophages.

**Conclusions:**

YQDZF inhibits the AKT/NF‐kB/HIF‐1α pathway, thereby mediating the M1 polarization of macrophages and the glycolytic metabolism to effectively alleviate the progression of POI.

**Abbreviations**
AKT: Protein Kinase B
ECAR: Extracellular Acidification
ELISA: Enzyme‐Linked Immunosorbent Assay
GLUT1: Glucose transporter 1
HIF‐1α: Hypoxia‐inducible factor 1α
HK2: Recombinant Hexokinase 2
H&E: Hematoxylin and eosin
IL‐1β: Interleukin‐1β
iNOS: Inducible Nitric Oxide Synthase
LA: 2‐Hydroxypropanoic acid
MPO: Myeloperoxidase
OCR: Oxygen Consumption Rate
p65, NF‐kB: Nuclear factor kappa‐B
PKM2: Pyruvate kinase isozyme typeM2
POI: Postoperative ileus
TNF‐α: Tumor Necrosis Factor‐alph
YQDZF: Yiqi Daozhi formula

## 1 Introduction

Postoperative ileus (POI) represents a gastrointestinal functional disorder that is an inevitable adverse consequence of surgical procedures [1]. Common symptoms include feelings of sickness, abdominal bloating, and a temporary cessation in bowel motility. Generally, the gastrointestinal function of postoperative patients can recover spontaneously within 3 days. However, some patients still experience prolonged gastrointestinal damage, which can progress to POI. The pathogenesis of POI is complex, with changes in gastrointestinal hormone levels, inflammatory cell activation, and electrolyte imbalances all considered as contributing factors [2]. The occurrence of POI not only affects the survival and prognosis of patients but also brings physical and economic distress. Currently, the clinical application of hormonal medications and prokinetic agents is the main treatment for POI, which has been proven effective in shortening the duration of POI. However, the strong side effects, significant risks, and high costs associated with these drugs affect their clinical use [3]. Hence, it is imperative to develop innovative clinical therapeutic strategies aimed at enhancing the treatment efficacy for POI.

It is generally accepted that the sustained phase of postoperative intestinal hypomotility due to bowel handling results from the inflammatory phase. More specifically, the prolonged dysmotility of the gastrointestinal tract associated with POI may result from the activation of the resident macrophages and the subsequent establishment of a neutrophilic infiltrate in the muscularis of the small intestine after bowel handling [4, 5]. Consequently, the inflammatory phase is frequently regarded as the primary target for intervention, and non‐steroidal anti‐inflammatory drugs (NSAIDs) have been widely applied to induce physiological motility [6]. Tissue‐resident macrophages are highly specialized phagocytes that carry out supportive functions during gastrointestinal development, homeostasis, and regeneration [7]. In the steady state, macrophages play a crucial role in protecting the host against harmful microorganisms and continuously phagocytose and clear luminal antigens that occasionally breach the epithelial layer [8]. In POI, macrophages directly drive the intestinal inflammation by excessive release of pro‐inflammatory cytokines [9].

Traditional Chinese medicine (TCM) is a well‐established medical system with a long history, which has shown great potential in treating functional gastrointestinal and motility disorders with minimal side effects [10, 11]. Yiqi Daozhi Formula (YQDZF) is a Chinese medicine prescription developed collaboratively by clinical and pharmacy experts from Affiliated Hospital of Nanjing University of Chinese Medicine, drawing upon extensive clinical experience. The formulation of YQDZF comprises 12 g Taizishen (*Pseudostellariae Radix*), 20 g Baizhu (*Atractylodis Macrocephalae Rhizoma*), 15 g Yunfuling (*Poria*), 20 g Yiyiren (*Coicis Semen*), 12 g Zhishi (Aurantii Fructus Immaturus), 10 g Houpo (*Magnoliae Officinalis Cortex*), 6 g Chenpi (*Citri Reticulatae Pericarpium*), 10 g Gancao (*Glycyrrhizae Radix et Rhizoma*).

In this formula, *Codonopsis pilosula* and *Atractylodes macrocephala* invigorate qi and strengthen the spleen to promote transformation and transportation; *bitter orange* and *magnolia bark* promote qi circulation and relieve bloating to relieve intestinal gas; *Poria cocos* and *Job's tears* both strengthen the spleen and remove dampness, enhancing the function of the monarch and minister herbs in supporting the spleen and promoting intestinal function; licorice harmonizes the other herbs. These herbs work together to support the righteous Qi and expel lingering pathogens, applying both attacking and tonifying methods to improve the postoperative patients’ resistance to disease and promote the early recovery of gastrointestinal function.

Our research was focused on investigating the precise molecular pathways that contribute to the therapeutic efficacy of YQDZF in the management of POI. First, we presented data indicating that the administration of YQDZF mitigated gut damage in mice with POI. Subsequently, our evidence showed that YQDZF significantly reduced the M1 polarization of macrophages and the glycolysis process. Lastly, in vitro cellular experiments confirmed that the functional mechanism was related to the AKT/NF‐kB/HIF‐1α signaling pathway in macrophages, which might be beneficial for the management of POI.

## 2 Materials and Methods

### 2.1 Experimental Animal Grouping and Treatment

SPF male C57BL/6 mice were purchased from Changzhou Kevins Laboratory Animal Co. LTD and were maintained under specific pathogen‐free conditions on a 12 h light/dark cycle and fed rodent chow and tap water ad libitum. All mice were adaptively fed for 1 week and were randomly divided into three groups based on a random sequence generated by a computer: control group with sham operation, POI group and YQDZF group (*n* = 8). The sample size of *n* = 8 per group was determined based on a review of similar experimental designs in the existing literature, as well as to adhere to ethical guidelines for animal use, aiming to minimize the number of animals while ensuring meaningful scientific outcomes [12, 13]. The exclusion criteria include (1) signs of unrelated illness, (2) serious surgical complications that would impede participation in the program, (3) died before the humane endpoint, and (4) technical errors or incomplete data. Body‐weight fluctuations and changes in defecation patterns in each experimental group were recorded.

### 2.2 Construction of the POI Mouse Model

The C57BL/6 mice in the POI and YQDZF groups were anesthetized and fixed in a supine position on the operating table. The abdomen was routinely depilated and disinfected. An incision of approximately 2 cm was made along the midline of abdomen. Sterilized ophthalmic forceps were used to dissect the small intestine. A wet cotton swab soaked in physiological saline was used to repeatedly wipe the small intestine for 4 min. After confirming the presence of congestion and edema, the small intestine was then returned to the abdominal cavity. The abdominal muscle layer and skin layer were sutured. The mice underwent laparotomy without intestinal manipulation were used as a Control group. The recovery of vitality and normal walking of mice confirmed the successful establishment of the POI model. Mice in the YQDZF group received approximately YQDZF (0.2 mL) by gavage, once a day (the equivalent ratio of mice to humans is 9.1; thus, 0.2 mL YQDZF is the clinical equivalent dosage of mice to humans). Mice in the POI and Control groups were administered with an equal volume of distilled water through gavage.

### 2.3 Preparation of YQDZF Solution

20 g each of *Rhizoma Atractylodis Macrocephalae* and *Semen Coicis* were weighed. 15 g of *Poria* were weighed. 12 g each of *Radix Pseudostellariae* and *Fructus Aurantii Immaturus* were weighed. 10 g each of *Radix Glycyrrhizae* and *Cortex Magnoliae Officinalis* were weighed. 6 g of *Pericarpium Citri Reticulatae* were weighed. The mixture was decocted in 500 mL of water, taken out 2 times every 50 min. After filtering, the solvent was evaporated using a rotary evaporator to a concentration of 1 kg/L and the product was dried by lyophilization.

### 2.4 Preparation of YQDZF‐Containing Serum

After a 7‐day acclimatization period, ten SPF‐grade male Sprague‐Dawley rats were randomly assigned to two experimental groups: the YQDZF group and the blank group, with five animals in each group. The rats in the YQDZF group were administered with YQDZF at a dose of 11 g/kg via gavage twice daily (morning and evening) for seven consecutive days. In contrast, the rats in the blank group received an equivalent volume of 0.9% distilled water. 1 h after the final gavage, the rats were anesthetized with 3% isoflurane, and blood was drawn from the abdominal aorta. Following a 60‐min incubation at room temperature, the blood samples were centrifuged at 3000 rpm for 15 min at 4°C. Subsequently, the supernatant was carefully decanted, inactivated by incubation in a 56°C water bath for 30 min, passed through a 0.22‐μm filter, and preserved at −80°C for future experimental analysis.

### 2.5 Liquid Chromatography‐Mass Spectrometry (LC‐MS/MS)

Analytes were separated by the Waters H‐Class UPLC system (Waters, USA) using a Waters CORTECS@ UPLCC18 (2.1 × 100 mm, 1.6 μm) at 30°C. The gradient solvent system consisted of acetonitrile (A) and 0.1% formic acid‐water (B) as follows: 0–5 min, 5%–10% A; 5–30 min, 10%–30% A; 30–45 min, 30%–50% A; 45–48 min, 50%–75% A; 48–51 min, 75%–95% A, which were delivered at a flow rate of 0.3 mL/min, UV detection at 190–400 nm, and an injection volume of 2 μL. MS data were acquired using the AB Sciex Triple TOF 4600 system (SCIEX, USA) equipped with an electrospray ionization (ESI) source. Analyte detection was carried out using MRM in a positive/negative mode.

### 2.6 Cell Culture

RAW264.7 cell line from National Collection of Authenticated Cell Cultures (Shanghai, China) was cultivated in high‐glucose DMEM supplemented with 10% fetal bovine serum and 1% penicillin/streptomycin within a controlled environment at 37°C in a humidified incubator at an atmosphere of 5% CO_2_. Regularly, the culture medium was renewed, and the cells were subcultured to ensure proper growth and health.

### 2.7 In Vitro POI Model Construction

As described by Mallesh et al. [14], RAW264.7 cells were cocultured with 100 ng/mL LPS + 20 ng/mL IFN‐γ in DMEM containing 10% FBS for 24 h.

### 2.8 Flow Cytometry Determination of Macrophage Surface Markers

After being prepared from the small intestines of mice, the single‐cell suspensions were incubated with Alexa Fluor® 647 anti‐mouse CD80 and Alexa Fluor® 488 anti‐mouse CD206 (104717, 141710, Bestopbio, China, Beijing) at room temperature for 30 min. The CALIBUR flow cytometer was used to detect cell surface markers.

### 2.9 ELISA

IL‐1β, TNF‐α, iNOS, MPO, LA, ECAR, and OCR ELISA kits were used to detect inflammatory factors, lactate accumulation, tissue myeloperoxidase activity, glycolysis, and consumption levels in tissue and cell samples. According to the kit instructions, the samples were reacted with HRP‐conjugated streptavidin and developed with the substrate TMB. The OD values were measured using a microplate reader (450 nm).

### 2.10 qRT‐PCR Detection

RT‐qPCR is mainly used to detect the gene expression of M1 and M2 markers after different grouping treatments. Total RNA was extracted using TRIzol reagent. The RNA was then reverse‐transcribed into cDNA, and the qRT‐PCR reaction conditions followed the manufacturer's instructions. The primers were synthesized by Genscript Biotech Co. Ltd. (China, Nanjing). The detailed information of the qRT‐PCR primers was shown in Table 1. β‐actin was used as the reference.

**Table 1:** Primers Required for the Experiment.

Primers	Forward	Reverse
IL‐6	5'‐TGCGTCCGTAGTTTCCTTCT‐3'	5'‐GCCTCAGACATCTCCAGTCC‐3'
TNF‐α	5'‐CCTCTCTCTAATCAGCCCTCTG‐3'	5'‐GAGGACCTGGGAGTAGATGAG‐3'
iNOS	5'‐CTCTTCGACGACCCAGAAAAC‐3'	5'‐CAAGGCCATGAAGTGAGGCTT‐3'
CD80	5'‐CCCCAGAAGACCCTCCTGAT‐3'	5'‐CCCGAAGGTAAGGCTGTTGTT‐3'
CD206	5'‐GGGTTGCTATCACTCTCTATGC‐3'	5'‐TTTCTTGTCTGTTGCCGTAGTT‐3'
ARG1	5'‐GTGGAAACTTGCATGGACAAC‐3'	5'‐AATCCTGGCACATCGGGAATC‐3'
β‐actin	5'‐GGAGCGAGATCCCTCCAAAAT‐3'	5'‐GGCTGTTGTCATACTTCTCATGG‐3'

### 2.11 Western Blot

RIPA Buffer (Cayman Chemical, State of Michigan, USA) was used to lyse cells and mice tissues. The total protein was transferred to SDS‐PAGE for electrophoresis for 120 min. Isolated proteins were transferred to the Immobilon‐E‐PVDF membrane (Merck, Darmstadt, Germany). The membrane was incubated with primary antibodies at 4°C for 12 h. The secondary antibodies were incubated for 2 h. The bands were developed using the ECL kit. Gray analysis was performed using the ImageJ 1.8.0 software. The antibodies used are shown below: anti‐GLUT1 (ab195021, Abcam, Cambridge, UK), anti‐HK2 (ab227198, Abcam), anti‐PKM2 (ab137791, Abcam), anti‐p65(ab76302, Abcam), anti‐AKT (ab8805, Abcam), anti‐p‐p65 (ab6503, Abcam), anti‐p‐AKT (ab8805, Abcam), anti‐HIF‐1α (ab179483, Abcam) and β‐actin (4967, CST).

### 2.12 Stabilization Assay

RAW264.7 cells were treated by Cycloheximide (Sigma‐Aldrich, St. Louis, MO) and MG‐132 (MedChemExpress, Monmouth Junction, NJ) at 0, 15, 30, 60 and 120 min. The protein expression levels of AKT were measured by Western blot.

### 2.13 Statistical Method

SPSS 20.0 statistical software was used for data analysis. Numerical data were expressed as the mean ± standard error of the mean. The data were tested for normal distribution, using a Shapiro‐Wilk test. Student's *t*‐test with chi‐square tests was used to compare categorical variables between two groups. One‐way ANOVA followed by Tukey's post hoc test was used to examine measurement data between three or more groups. *p* < 0.05 indicated a significant difference.

## 3 Results

### 3.1 YQDZF Improves POI Symptoms in Mice

**Figure 1:**
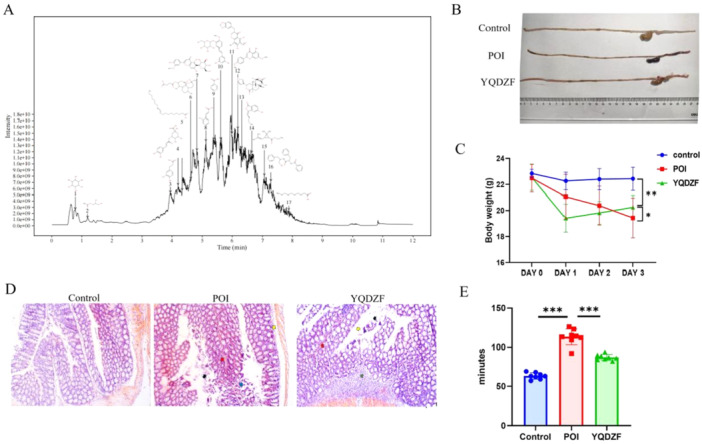
YQDZF alleviates the symptoms of POI mice. (A) The fingerprint of YQDZF in LC‐MS/MS analysis. (B) Intestinal morphology map. (C) Changes in body weight of mice. (D) HE staining of intestinal pathological images of mice in different groups. (E) Carmine detection of intestinal motility. *p < 0.05; **p < 0.01. *n* = 8, one‐way analysis of variance was utilized to compare difference among multiple groups, followed by Tukey post hoc test.

As shown in the LC‐MS/MS characterization (Figure 1A), myo‐Inositol, 2,3‐Dihydroxypropyl acetate, 3,4‐Dihydroxyhydrocinnamic acid, Chlorogenic acid, Cis‐10‐Nonadecenoic_acid, Artesunate, Isoscoparin, 2‐Hydroxycinnamic acid, Benzoic acid, (2S,3 R,4S,5S,6 R)‐2‐[5‐[(E)‐2‐(3,5‐dihydroxyphenyl)vinyl]‐2‐methoxy‐phenoxy]‐6‐(hydroxymethyl)tetrahydropyran‐3,4,5‐triol, Hemerocallone, 7,4′‐Dimethoxy‐5‐hydroxyisoflavone, Columbin, (3Z)‐3‐butylidene‐5‐hydroxy‐isobenzofuran‐1‐one, ethyl 2,2‐dimethyl‐3‐(2‐methylprop‐1‐enyl)cyclopropanecarboxylate, Aurantiamide acetate and Capric acid were the most enriched active ingredients in YQDZF (Table 2). To investigate whether YQDZF was effective in treating POI mice, we initially investigated the impact of YQDZF on the intestinal length in the POI mouse model. Ileum and colon of the POI group mice exhibited significant edema, accompanied by adhesions and congestion, which were improved by YQDZF (Figure 1B). Furthermore, at day 1, YQDZF treatment significantly reduced mouse body weight, which might be related to the effects of YQDZF on relieving obstruction and reducing edema and its potential side effects, such as diarrhea and loss of appetite. However, the use of YQDZF also significantly increased the body weight of the POI mice from day 1 to day 3 (Figure 1C). Moreover, the severe intestinal damage in POI mice was mitigated by YQDZF (Figure 1D). The initial defecation timing in POI mice was markedly delayed compared to that of Control group and YQDZF group (Figure 1E). Taken together, YQDZF treatment markedly enhanced gut function in a POI mouse model, which included alleviating intestinal edema and adhesions, restoring weight gain, reducing intestinal tissue injury, and shortening fecal transit time.

**Table 2:** The information of the identified compounds in YQDZF extract by LC‐MS/MS.

No.	Name	Formula	Rt/s	Measured (m/z)
1	myo‐Inositol	C_6_H_12_O_6_	44.7	179.0558 [M‐H]‐
2	2,3‐Dihydroxypropyl acetate	C_5_H_10_O_4_	70.3	133.0503 [M‐H]‐
3	3,4‐Dihydroxyhydrocinnamic acid	C_9_H_10_O_4_	234.9	181.0503 [M‐H]‐
4	Chlorogenic acid	C_16_H_18_O_9_	250.9	353.0873 [M‐H]‐
5	Cis‐10‐Nonadecenoic_acid	C_19_H_36_O_2_	260.5	277.1553 [M‐H]‐
6	Artesunate	C_19_H_28_O_8_	276.7	383.1703 [M‐H]‐
7	Isoscoparin	C_22_H_22_O_11_	289.5	461.1082 [M‐H]‐
8	2‐Hydroxycinnamic acid	C_9_H_8_O_3_	307.7	163.0399 [M‐H]‐
9	Benzoic acid	C_7_H_6_O_2_	323.1	121.0292 [M‐H]‐
10	(2S,3 R,4S,5S,6 R)‐2‐[5‐[(E)‐2‐(3,5‐dihydroxyphenyl)vinyl]‐2‐methoxy‐phenoxy]‐6‐(hydroxymethyl)tetrahydropyran‐3,4,5‐triol	C_21_H_24_O_9_	336.2	419.1334 [M‐H]‐
11	Hemerocallone	C_19_H_16_O_7_	359.4	355.0817 [M‐H]‐
12	7,4'‐Dimethoxy‐5‐hydroxyisoflavone	C_17_H_14_O_5_	370.9	297.0763 [M‐H]‐
13	Columbin	C_20_H_22_O_6_	379	393.1101 [M+Cl]‐
14	(3Z)‐3‐butylidene‐5‐hydroxy‐isobenzofuran‐1‐one	C_12_H_12_O_3_	399.5	203.071 [M‐H]‐
15	ethyl 2,2‐dimethyl‐3‐(2‐methylprop‐1‐enyl)cyclopropanecarboxylate	C_12_H_20_O_2_	423.6	195.1387 [M‐H]‐
16	Aurantiamide acetate	C_27_H_28_N_2_O_4_	436.5	425.184 [M‐H2O‐H]‐
17	Capric acid	C_10_H_20_O_2_	473.1	171.1387 [M‐H]‐

### 3.2 YQDZF Affects the Balance between M1 and M2 Macrophages in POI Mice

**Figure 2:**
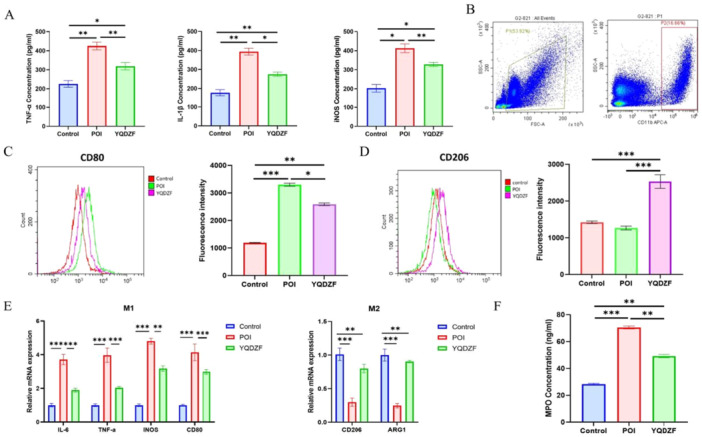
YQDZF affects the balance between M1 and M2 macrophages in POI mice. (A) The protein levels of IL‐1β, TNF‐α and iNOS were detected by ELISA. (B) Total macrophages were sorted by flow cytometry. (C, D) Flow cytometry sorting of M1 and M2 macrophages. (E) qRT‐PCR was used to detect the gene expression of M1 markers (IL‐6, TNF‐α, iNOS and CD80) and M2 markers (CD206 and ARG1). (F) The expression level of MPO in tissues was detected by ELISA. *p < 0.05; **p < 0.01; ***p < 0.001. *n* = 8, one‐way analysis of variance was utilized to compare difference among multiple groups, followed by the Tukey post hoc test.

ELISA measured the inflammatory markers (IL‐1β, TNF‐α, and iNOS) and the results demonstrated that YQDZF notably suppressed the elevated levels of these inflammatory mediators in the POI mice model (Figure 2A). Next, we used flow cytometry to sort the single‐cell suspension of the small intestine. Monocytes were sorted from the total cells, and then total macrophages‐, CD11b‐positive cells, were sorted (Figure 2B). Further analysis of the expression levels of M1 and M2 macrophage polarization markers was conducted. The results showed that the expression level of CD80 in the POI group of mice was significantly increased compared with the control group; however, the expression of CD206 was decreased (Figure 2C,D). Analysis of qRT‐PCR data revealed a marked increase in the levels of IL‐6, TNF‐α, iNOS, and CD80 in the cellular suspension obtained from the small intestine of mice in the POI group. The addition of YQDZF significantly inhibited IL‐6, TNF‐α, iNOS, and CD80 levels. The expression levels of CD206 and ARG1 expression were notably decreased in the POI group, which were promoted by the application of YQDZF (Figure 2E). Also, a marked elevation in the concentration of MPO was observed in the tissues of POI mice. The application of YQDZF reduced the expression of MPO (Figure 2F). In conclusion, our findings suggested that the application of YQDZF could significantly exert anti‐inflammatory effects in POI mice by suppressing the M1 phenotype polarization of macrophages.

### 3.3 YQDZF Inhibits Aerobic Glycolysis Metabolism in POI Mice

**Figure 3:**
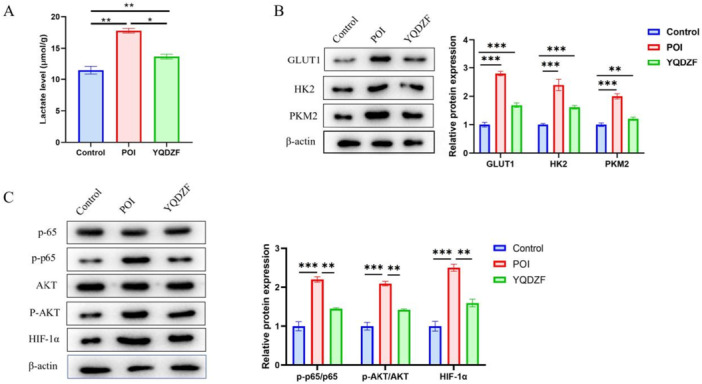
YQDZF inhibits aerobic glycolysis metabolism in POI mice. (A) ELISA was used to detect lactate levels. (B, C) Western blot was used to detect the protein levels of GLUT1, HK2, PKM2, p65, AKT, p‐p65, p‐AKT, and HIF‐1α. *p < 0.05; **p < 0.01; ***p < 0.001. *n* = 8, one‐way analysis of variance was utilized to compare difference among multiple groups, followed by Tukey post hoc test.

To further elucidate the therapeutic effect of YQDZF on POI, we analyzed its impact on the glycolysis level in the model mice. The findings from the ELISA assays revealed that the concentration of lactate was enhanced in the POI group. In comparison, the YQDZF group exhibited a notably reduced lactate level in comparison with the POI group (Figure 3A). The levels of GLUT1, HK2, and PKM2 proteins in the intestinal tissue from the POI group were increased by contrast with both the Control and YQDZF group (Figure 3B). Utilizing Western blot analysis to examine the AKT/NF‐kB/HIF‐1α signaling pathway, the results indicated elevated protein levels of p‐p65, p‐AKT, and HIF‐1α in the POI group, which were higher in both the Control and YQDZF group (Figure 3C). Based on the above results, we proposed the hypothesis that YQDZF mediated the AKT/NF‐kB/HIF‐1α pathway to inhibit lactate accumulation in POI mice, which might affect the M1 polarization of macrophages.

### 3.4 YQDZF Mediates the Inhibition of Aerobic Glycolysis Metabolism in Macrophages of POI Mice through the AKT/NF‐kB/HIF‐1α Pathway

**Figure 4:**
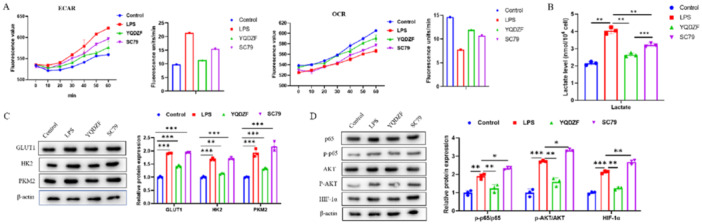
The AKT/NF‐kB/HIF‐1α pathway affects metabolism in RAW264.7 cells. (A) ELISA was used to detect glycolysis (ECAR) and oxygen consumption (OCR), and fluorescence values and slope statistics were recorded. (B) ELISA was used to detect lactate levels. (C, D) Western blot was used to detect the protein levels of GLUT1, HK2, PKM2, p65, AKT, p‐p65, p‐AKT, and HIF‐1α. *p < 0.05; **p < 0.01; ***p < 0.001. *n* = 3, one‐way analysis of variance was utilized to compare difference among multiple groups, followed by Tukey post hoc test.

To validate this scientific hypothesis, we analyzed the glycolytic level of RAW264.7 cells through in vitro experiments. RAW264.7 cells were treated with LPS or SC79 (AKT agonist). As depicted in Figure 4A, the ECAR levels in the LPS group were markedly elevated and the OCR levels were decreased compared to the Control group, while YQDZF down‐regulated ECAR levels and up‐regulated OCR levels. ECAR levels in the SC79 group were significantly higher, and OCR levels were lower than that of YQDZF group (Figure 4B). Meanwhile, the levels of GLUT1, HK2, and PKM2 protein expression in the YQDZF group were considerably reduced compared to the LPS and SC79 groups (Figure 4C). p‐p65/p65, p‐AKT/AKT, and HIF‐1α protein levels were enhanced in the LPS group. Compared to the LPS and SC79 groups, the YQDZF group demonstrated reduced protein abundance of p‐p65/p65, p‐AKT/AKT, and HIF‐1α (Figure 4D). The above cellular experiments confirmed that AKT was a key factor for YQDZF to inhibit aerobic glycolysis in POI macrophages.

### 3.5 YQDZF Enhances AKT Proteasome Degradation

**Figure 5:**
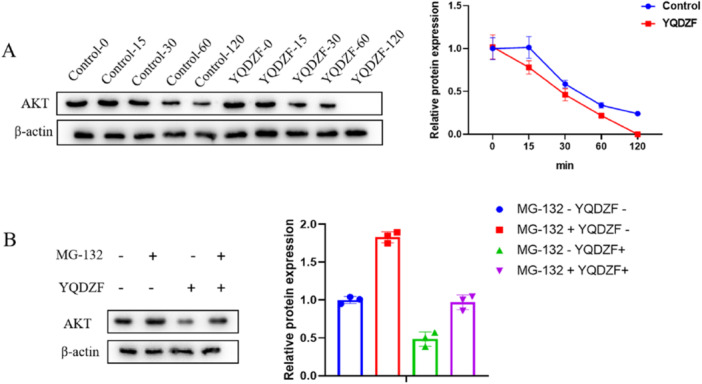
YQDZF inhibits glycolysis by enhancing the proteasomal degradation of AKT. (A, B) The protein stability of AKT was determined by the cycloheximide and MG‐132 assay. **p < 0.01; ***p < 0.001. *n* = 3, one‐way analysis of variance was utilized to compare difference among multiple groups, followed by Tukey post hoc test.

To delve into the precise mechanism through which YQDZF suppressed the AKT/NF‐κB/HIF‐1α signaling pathway, CHX and MG‐132 were employed to assess the stability of the AKT protein. After CHX treatment, YQDZF group showed significant degradation of AKT protein (Figure 5A). The enrichment level of AKT protein was increased after MG‐132 treatment, which was reduced by YQDZF (Figure 5B). This indicated that YQDZF had a significant effect on the degradation of AKT proteasome.

### 3.6 YQDZF Promotes Macrophage Polarization Towards M2 by Glycolytic Metabolism

**Figure 6:**
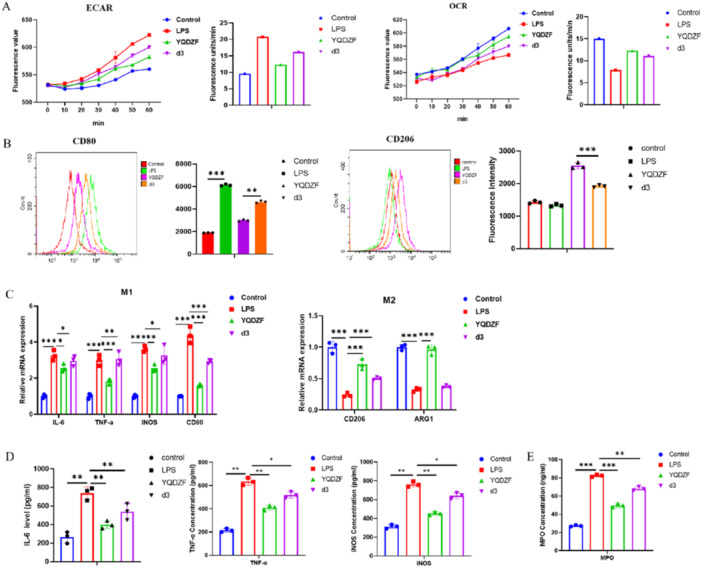
YQDZF affects RAW264.7 polarization through metabolism. (A) ELISA was used to detect glycolysis (ECAR) and oxygen consumption (OCR), and fluorescence values and slope statistics were recorded. (B) Flow cytometry for M1 and M2 type macrophages. (C) qRT‐PCR was used to detect the gene expression of M1 and M2 macrophage markers. (D, E) ELISA was used to detect the protein levels of IL‐6, TNF‐α, iNOS, and MPO. *p < 0.05; **p < 0.01; ***p < 0.001. *n* = 3, one‐way analysis of variance was utilized to compare difference among multiple groups, followed by Tukey post hoc test.

To elucidate the relationship between YQDZF‐mediated inhibition of AKT/NF‐κB/HIF‐1α pathway, aerobic glycolysis in POI macrophages, and macrophage polarization, Fenbendazole‐d3 (d3) was used to promote the activation of HIF‐1α. The findings from the ELISA tests indicated that the application of YQDZF considerably decreased the ECAR level and increased the OCR level in the LPS‐induced RAW264.7 cell model. The addition of d3 significantly increased the ECAR level while decreasing the OCR level (Figure 6A). Flow cytometry analysis revealed that the application of YQDZF markedly diminished the levels of CD80 expression while concomitantly elevating the levels of CD206 expression. The addition of d3 reversed this result (Figure 6B). The findings from qRT‐PCR analyses indicated a marked reduction in the expression of IL‐6, TNF‐α, iNOS, and CD80 and an elevation in the expression of CD206 and ARG1 in the YQDZF group when compared to the LPS group. The d3 group exhibited notably enhanced IL‐6, TNF‐α, iNOS, and CD80 expression and declined CD206, ARG1 expression compared to the YQDZF group (Figure 6C). Meanwhile, ELISA analyses for inflammatory cytokines indicated a marked increase in IL‐6, TNF‐α, and iNOS levels in the d3 group as compared to the YQDZF group (Figure 6D). Ultimately, the MPO expression in the YQDZF group was substantially reduced in comparison to the d3 group (Figure 6E). In summary, YQDZF inhibited macrophage polarization towards M1 by glycolytic metabolism.

## 4 Discussion

Numerous clinical trials have validated the preliminary efficacy of TCM for addressing post‐surgical complications [15, 16, 17], which can not only alleviate inflammation but also effectively reduce the clinical risks posed by complications. Our investigation was focused on elucidating the precise mode of action of YQDZF in the management of POI. We found that YQDZF effectively relieved POI in mouse models, the mechanism of which might be related to the balance of macrophage M1 and M2. *In vitro* experiments confirmed that YQDZF mediated the AKT/NF‐κB/HIF‐1α pathway to regulate glycolysis metabolism and inhibit the polarization towards M1 macrophages.

POI is a postoperative complication commonly seen after abdominal surgery with a complex etiology. Its clinical manifestations mainly include abdominal pain, bloating, and cessation of anal exhaust and defecation [18]. Severely, some POI patients may even suffer from postoperative ileal effusion and expansion, as well as dysbiosis and translocation of the flora. Fan et al. have confirmed the significant correlation between inflammatory biomarkers and POI through a clinical big data prediction model [19]. Recently, studies have validated that conventional Chinese medical interventions, including practices like acupuncture and the administration of Da‐Cheng‐Qi‐Tang, serve as efficacious clinical approaches in aiding the rehabilitation of patients with POI [20, 21]. It is composed of various traditional Chinese herbs. Studies by Zhang et al. and Qin et al. have mentioned that *Radix Pseudostellariae* and *Atractylodes macrocephala* that primarily function to replenish Qi and strengthen the spleen are able to enhance spleen and stomach functions [22, 23]. Qiao et al. and Ma et al.‘s work suggests that *Citrus aurantium* and *Magnolia officinalis* can alleviate gastrointestinal bloating [24, 25]. *Poria cocos* and *Coix lacryma‐jobi* have been confirmed to relieve gastrointestinal inflammation and protect the spleen [26, 27, 28]. Preliminarily, YQDZF was found to improve the surgical outcome, intestinal edema, adhesion and congestion, increase body weight and mitigate intestinal damage in POI mice.

Khawaja and colleagues have highlighted that the inflammatory response plays a crucial role in the development of POI [29]. Our study also confirmed the activated inflammatory reaction, as evidenced by the abnormally elevated levels of proinflammatory cytokines, including IL‐1β, TNF‐α, and iNOS in the POI mouse model. Notably, the active ingredients that have been identified in YQDZF have been widely reported to play the anti‐inflammatory roles [30, 31, 32, 33, 34, 35, 36]. Our present findings expectedly demonstrated the anti‐inflammatory role of YQDZF in POI mice. Macrophages exhibit remarkable plasticity in terms of their function and phenotype and can polarize into different subpopulations, including the proinflammatory M1 and profibrotic M2 macrophages, functioning as primary regulators of inflammation induction and resolution [37]. The M1 phenotype is stimulated by IFN‐γ, TNF, and TLR ligands, releasing relatively high levels of proinflammatory cytokines such as IL‐1, IL‐6, and TNF‐α. Conversely, the M2 phenotype can be induced by stimulation with IL‐4, IL‐10, or IL‐13, releasing relatively high levels of anti‐inflammatory cytokines such as IL‐10 and TGF‐β1. Mazzotta et al. have also mentioned that macrophages can serve as a primary therapeutic target for POI [38], and emerging evidence has demonstrated that the imbalance of M1/M2 polarization is involved in intestinal inflammation during the process of POI [39, 40]. In particular, the active ingredients of YQDZF have been reported to participate in macrophage function and polarization. For example, Myo‐Inositol in Fermented Papaya Preparation can improve human macrophage function [41]. 3,4‐dihydroxyphenylpropionic acid, as the gut microbial metabolite, can suppress macrophage pro‐inflammatory activation in hepatic ischemia/reperfusion injury [42]. Chlorogenic acid reprograms macrophage activation in sepsis‐induced acute lung injury and glioblastoma [43, 44]. Artesunate can also inhibit M1 macrophage polarization in sepsis‐induced liver injury and atherosclerosis [45, 46]. On the basis of the aforementioned components, our research established both in vivo and in vitro POI models and validated the suppressive role of YQDZF on the M1 polarization of macrophages through reducing M1 macrophage markers IL‐6, TNF‐α, iNOS, and CD80 expression while raising M2 macrophage markers CD206 and ARG1 expression, suggesting the potent anti‐inflammatory role of YQDZF during POI via repolarizing macrophages from M1 to M2 phenotype. This provides new evidence for the main target of inhibiting POI occurrence proposed by Wang et al. and Pohl et al., which is the activation of intestinal muscularis macrophages mediating inflammatory responses [40, 47]. MPO, a heme‐containing peroxidase expressed mainly in neutrophils, has been demonstrated to be a local mediator of tissue damage and the resulting inflammation in various inflammatory diseases [48]. Also, YQDZF was also observed to decrease MPO activity in POI mice.

Aerobic glycolysis is the main metabolic pathway of M1 macrophage polarization, with lactate as the primary metabolic product. Tao et al. have confirmed that through omics analysis, this mechanism effectively promotes the rapid response of M1 macrophages to external stimuli and plays a pro‐inflammatory role in the inflammatory environment of colitis [49]. Zhang et al. have proposed that Xiang Lian Wan inhibits M1 macrophage polarization through metabolic reprogramming of aerobic glycolysis, thereby reducing the inflammatory response in ulcerative colitis [50]. Our research confirmed that YQDZF down‐regulated lactate levels and GLUT1, HK2, and PKM2 expression in POI mice, implying that YQDZF inhibited the glycolysis of M1‐polarized macrophages. In terms of molecular mechanisms, the AKT pathway converges inflammatory and metabolic signals to regulate macrophage responses, modulating their activation phenotype [51]. Duan et al. have proposed that through pharmacological and animal model studies, Kuanchang‐Shu granule regulates AKT to protect against POI in rats [52]. The AKT protein can initiate the activation of IKB kinase (IKKα), leading to the degradation of the NF‐κB inhibitor IκB [53]. This process ultimately results in the release of NF‐κB from the cytoplasm, translocation into the nucleus, and the subsequent transcription of its target genes [54]. Moreover, HIF‐1α induction is disrupted in the absence of NF‐κB activity, even under sustained hypoxic conditions, suggesting that HIF‐1α transcription is predominantly governed by NF‐κB [55]. Interestingly, HIF‐α can upregulate key enzymes in the glycolytic pathway, such as hexokinase, phosphofructokinase, and lactate dehydrogenase, thereby increasing glycolytic flux and affecting cellular energy metabolism [56]. Liu et al.‘s research has confirmed that Shenhuang plaster mediates the AKT/NF‐κB axis to alleviate POI symptoms and inflammation [57]. Wu et al.‘s research has proposed that MDM2, by activating HIF‐1α, enhances the inflammation and glycolysis of M1 macrophages [58]. Our findings for the first time confirmed that YQDZF decreased p‐p65/p65, p‐AKT/AKT and HIF‐1α in POI mice. In vitro, the declined ECAR levels, the increased OCR levels, the reduced lactate levels, the repressed GLUT1, HK2, PKM2, p‐p65/p65, p‐AKT/AKT and HIF‐1α expression imposed by YQDZF were all reversed by SC79, a novel and specific AKT activator, suggesting that the AKT/NF‐κB/HIF‐1α axis was highly related to the glycolytic metabolism mediated by YQDZF in POI. Also, we accidentally found that YQDZF significantly degraded AKT protein after CHX treatment and reduced AKT protein enrichment after MG‐132 treatment. Based on this, we believe that YQDZF may regulate the AKT/NF‐κB/HIF‐1α axis by promoting the proteasomal degradation of AKT. Furthermore, the HIF‐1 pathway agonist, d3, partially reversed the suppressive role of YQDZF in M1 macrophage polarization and the promoting role in M2 macrophage polarization in vitro. Combined with these findings, the therapeutic mechanism of YQDZF in alleviating POI was dependent on the inhibition of M1 macrophage inflammation and glycolysis via the AKT/NF‐κB/HIF‐1α axis. Based on the findings of this study, we propose that the administration of YQDZF restores intestinal immune‐metabolic homeostasis by modulating macrophage polarization and glycolytic metabolism. This effectively alleviates the symptoms of POI, such as intestinal obstruction and weight loss. Therefore, YQDZF holds potential for clinical translation.

Due to the complexity mechanism of POI, YQDZF may be involved in macrophage polarization in POI via other pathways besides AKT/NF‐κB/HIF‐1α signaling pathway. Besides, considering a mouse model is limited in many respects compared to a clinical study, the effects of YQDZF on POI patients, including the long‐term effect, possible side effects, and protective rate, also warrant to be evaluated. Accordingly, future research will be dedicated to a more thorough assessment of YQDZF's potential mechanisms of action, including: ① a systematic analysis of YQDZF's components using bioinformatics tools and high‐throughput screening to predict potential targets and construct a component‐target network for a more complete understanding of its mechanism; ② investigation of whether YQDZF influences other pathways related to macrophage polarization and glycolysis metabolism beyond the AKT/NF‐kB/HIF‐1α pathway, such as STAT3 and MAPK; and ③ validation of YQDZF's specific effects on selected targets using gene knockout and RNA interference techniques to eliminate non‐specific effects.

## 5 Conclusion

In brief, our study is the first to put forward YQDZF and innovatively analyze its therapeutic effects on POI and the specific mechanisms involved. Macroscopically, YQDZF was effective in improving pathological damage and promoting intestinal function in a POI mouse model. This therapeutic effect was traced to the cellular and immune levels, where the formula was found to suppress M1 macrophage polarization and subsequent inflammation. Mechanistically, we demonstrated that YQDZF achieved this immunomodulation by inhibiting aerobic glycolysis in M1 macrophages. This metabolic regulation, in turn, was controlled via the suppression of the AKT/NF‐κB/HIF‐1α signaling pathway. Our investigation may establish a fresh fundamental theoretical framework for the application of TCM in mitigating POI.

## Author Contributions

Conceptualization, Wang Gang and Jiang Zhiwei; methodology, Wang Gang, Jiang Zhiwei and Tao Yuewei; validation, Wang Gang, Jiang Zhiwei, Zhao Xuan and Yi Chen; formal analysis, Wang Ye and Yu Jing; investigation and data curation, Wang Gang, Zhang Tianle and Shao Mingyue; writing—original draft preparation and editing, Wang Gang; funding acquisition, Jiang Zhiwei. All authors have read and agreed to the published version of the manuscript.

## Ethics Statement

All animal experiments were approved by the Ethics Committee of Affiliated Hospital of Nanjing University of Chinese Medicine (2024DW‐061‐01).

## Conflicts of Interest

The authors declare no conflicts of interest.

## Data Availability

The datasets generated and/or analyzed during the current study are available from the corresponding author on reasonable request.
